# Elevated De Ritis Ratio Is Associated With Poor Prognosis in COVID-19: A Systematic Review and Meta-Analysis

**DOI:** 10.3389/fmed.2021.676581

**Published:** 2021-12-22

**Authors:** Raymond Pranata, Ian Huang, Michael Anthonius Lim, Emir Yonas, Rachel Vania, Antonia Anna Lukito, Sally Aman Nasution, Bambang Budi Siswanto, Raden A. Tuty Kuswardhani

**Affiliations:** ^1^Faculty of Medicine, Universitas Pelita Harapan, Tangerang, Indonesia; ^2^Faculty of Medicine, Department of Internal Medicine, Hasan Sadikin General Hospital, Universitas Padjadjaran, Bandung, Indonesia; ^3^Faculty of Medicine, Universitas YARSI, Jakarta, Indonesia; ^4^Faculty of Medicine, Division of Plastic, Reconstructive, and Aesthetic, Department of Surgery, Sanglah General Hospital, Udayana University, Jimbaran, Indonesia; ^5^Department of Cardiology and Vascular Medicine, Siloam Hospitals Lippo Village, Tangerang, Indonesia; ^6^Faculty of Medicine, Division of Cardiology, Department of Internal Medicine, Universitas Indonesia/Cipto Mangunkusumo National General Hospital, Jakarta, Indonesia; ^7^Faculty of Medicine, Department of Cardiology and Vascular Medicine, National Cardiovascular Center Harapan Kita, Universitas Indonesia, Jakarta, Indonesia; ^8^Faculty of Medicine, Division of Geriatrics, Department of Internal Medicine, Sanglah Teaching Hospital, Udayana University, Denpasar, Indonesia

**Keywords:** coronavirus—COVID-19, liver enzyme, transaminase, SARS-CoV-2, De Ritis ratio

## Abstract

**Objective:** This meta-analysis aims to assess whether elevated De Ritis ratio is associated with poor prognosis in patients with coronavirus 2019 (COVID-19).

**Methods:** A systematic literature search was performed using PubMed, Embase, and EuropePMC databases up until September 17, 2021. De Ritis ratio is also known as Aspartate aminotransferase/alanine transaminase (AST/ALT) ratio. The main outcome was poor prognosis, a composite of mortality, severity, the need for ICU care, and intubation. The effect measure was odds ratios (ORs) and mean differences. We generated sensitivity and specificity, negative and positive likelihood ratio (NLR and PLR), diagnostic odds ratio (DOR), and area under curve (AUC).

**Results:** There were eight studies with 4,606 patients. De Ritis ratio was elevated in 44% of the patients. Patients with poor prognosis have higher De Ritis ratio [mean difference 0.41 (0.31, 0.50), *p* < 0.001; *I*^2^: 81.0%] and subgroup analysis showed that non-survivors also have higher De Ritis Ratio [mean difference 0.47 (0.46, 0.48), *p* < 0.001; *I*^2^: 0%]. Elevated De Ritis ratio was associated with poor prognosis [OR 3.28 (2.39, 4.52), *p* < 0.001; *I*^2^: 35.8%]. It has a sensitivity of 55% (36–73), specificity of 71% (52–85), PLR 1.9, NLR.63, DOR of 3 (2–4), and AUC of.67 (0.63–0.71). The posterior probability of poor prognosis was 38% if De Ritis is elevated, while 17% if De Ritis is not elevated.

**Conclusion:** Elevated De Ritis ratio is associated with poor prognosis in patients with COVID-19.

**Systematic Review Registration:** PROSPERO ID: CRD42020216634.

## Introduction

The severe acute respiratory syndrome coronavirus 2 (SARS-CoV-2) spread rapidly and causes a considerable number of deaths worldwide (1). Although most patients with coronavirus 2019 disease (COVID-19) have mild-to-moderate symptoms, they may develop severe COVID-19 with multi-organ dysfunction, cardiorespiratory collapse, coagulopathy and thrombosis, sepsis, and even death (2, 3). Common symptoms include fever, cough and dyspnea, and minor symptoms are dysgeusia, anosmia, gastrointestinal symptoms, cutaneous manifestation, and headache (4–6). Although the virus primarily affects the lungs, it may invade and damage other organs, such as the heart and vasculature, coagulation system, liver, kidneys, intestine, and central nervous system (7–12).

Severe acute respiratory syndrome coronavirus 2 (SARS-CoV-2) has been reported to cause a varying degree of liver injury (13). Liver injury is more frequently found in patients with severe COVID-19 and is associated with an increased risk of poor outcomes (14). The ratio between the two most routinely requested liver function panel, the aspartate aminotransferase (AST)/alanine aminotransferase (ALT) ratio or more commonly known as the De Ritis ratio, was recently reported as a possible biomarker for prognostication in patients with COVID-19 (15). Therefore, we conducted a systematic review and meta-analysis to evaluate the association between De Ritis ratio and composite poor outcomes in COVID-19.

## Materials and Methods

The study was registered in the PROSPERO database (CRD42020216634) and was conducted per the Preferred Reporting Items for Systematic Reviews and Meta-Analyses (PRISMA) guidelines.

### Eligibility Criteria

Research articles (both prospective and retrospective cohorts) that contain information on De Ritis ratio and mortality, severity, intensive care unit (ICU) care admission or need for intubation were included in the study. We excluded preprints, review articles, editorial, commentaries, conference abstracts, letters, and case reports/series.

### Search Strategy and Study Selection

We performed a systematic literature search from PubMed database, Embase database, and EuropePMC database with the search terms “COVID-19” OR “2019-nCoV” OR “SARS-CoV-2” AND “De Ritis Ratio” OR “AST ALT Ratio.” The search was finalized on September 17, 2021. The PubMed search strategy was [(COVID-19) OR (2019-nCoV) OR (SARS-CoV-2)] AND [(De Ritis Ratio) OR (AST ALT Ratio)]. Two independent authors performed the initial search and duplicate removal. The inclusion and exclusion criteria served as the basis for article exclusion during the title or abstract screening and evaluation of full-text articles.

### Data Collection

Data extraction from the eligible studies was conducted by two authors who are independently using pre-built forms containing the author, study design, origin, AST, ALT, cut-off for elevated De Ritis ratio, sample size, age, gender, obesity, diabetes, elevated liver enzymes, and outcome of interests.

The main outcome was poor prognosis, a composite of mortality, severity, need for ICU care, and need for intubation. Mortality was defined as non-survivor or death.

Severity was defined according to the studies inclusion parameters, need for ICU care, and intubation. The effect measure was the odds ratios (ORs) and mean differences. Diagnostic meta-analysis was performed to generate diagnostic values, which consisted of sensitivity, specificity, negative and positive likelihood ratio (NLR and PLR), diagnostic odds ratio (DOR), and area under curve (AUC).

### Risk of Bias Assessment

The risk of bias assessment was performed independently by two authors with the help of Newcastle-Ottawa Scale (NOS). Discrepancies were resolved by discussion. The Egger's test and Deek's funnel plot asymmetry test was used to assess the presence of small-study effects and publication bias, respectively.

### Statistical Analysis

STATA 16 (College Station, TX) was used to perform statistical analysis. Meta-analysis of proportions was performed to pool the incidence of elevated De Ritis Ratio. DerSimonian and Laird method random-effects models were used to pool ORs and mean differences, notwithstanding heterogeneity. *p* < 0.05 were considered statistically significant. Inter-study heterogeneity was evaluated using the I-squared (*I*^2^) and Cochrane *Q* test, an *I*^2^ > 50% or *p* < 0.10 indicates substantial heterogeneity. We performed pooling of sensitivity and specificity and generated a summary receiver operating characteristic (SROC) curve. Relationship between prior probability and posterior probability was evaluated using Fagan's nomogram. Subgroup analysis was performed for mortality outcome.

## Results

### Baseline Characteristics

**Figure 1:**
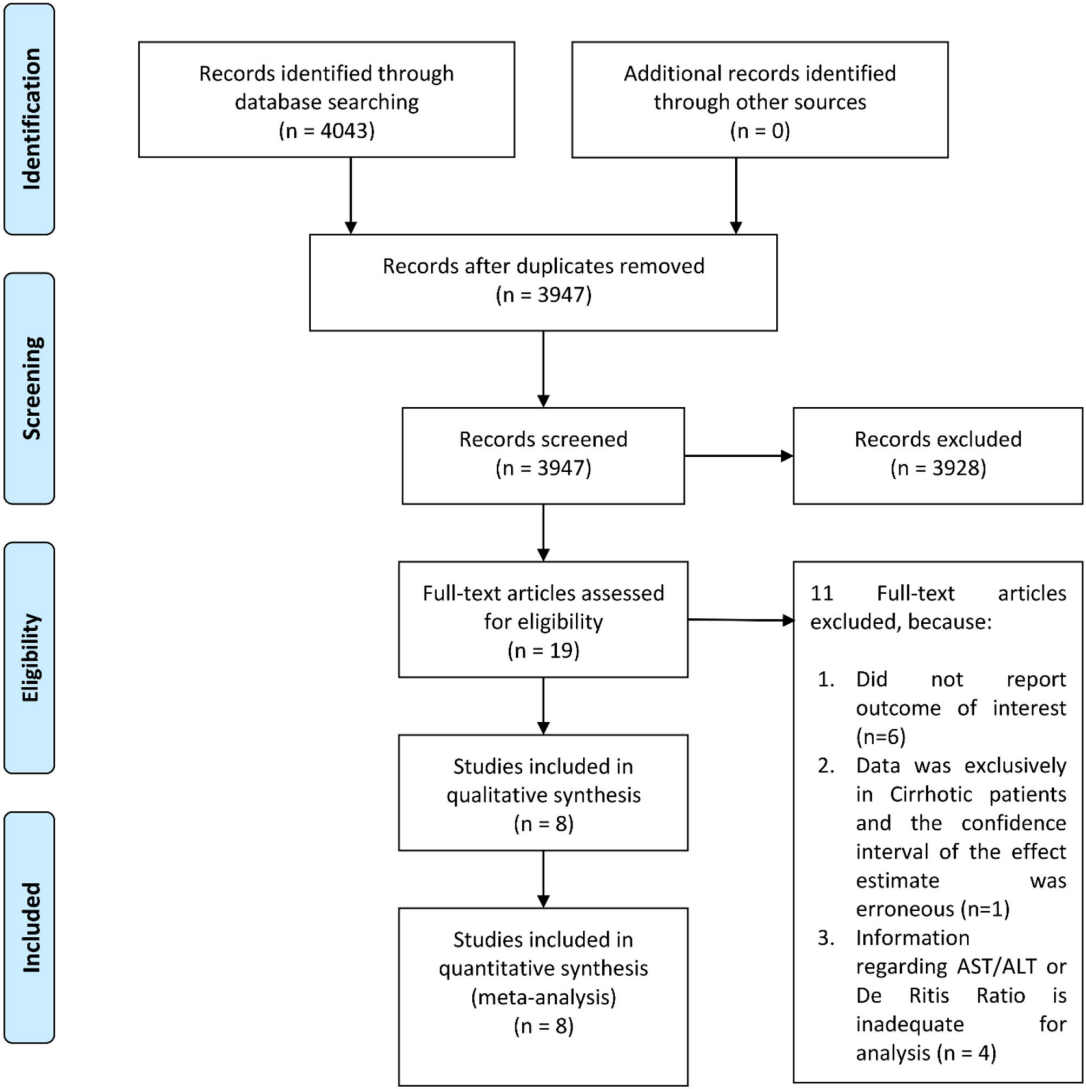
PRISMA flowchart.

**Figure 2:**
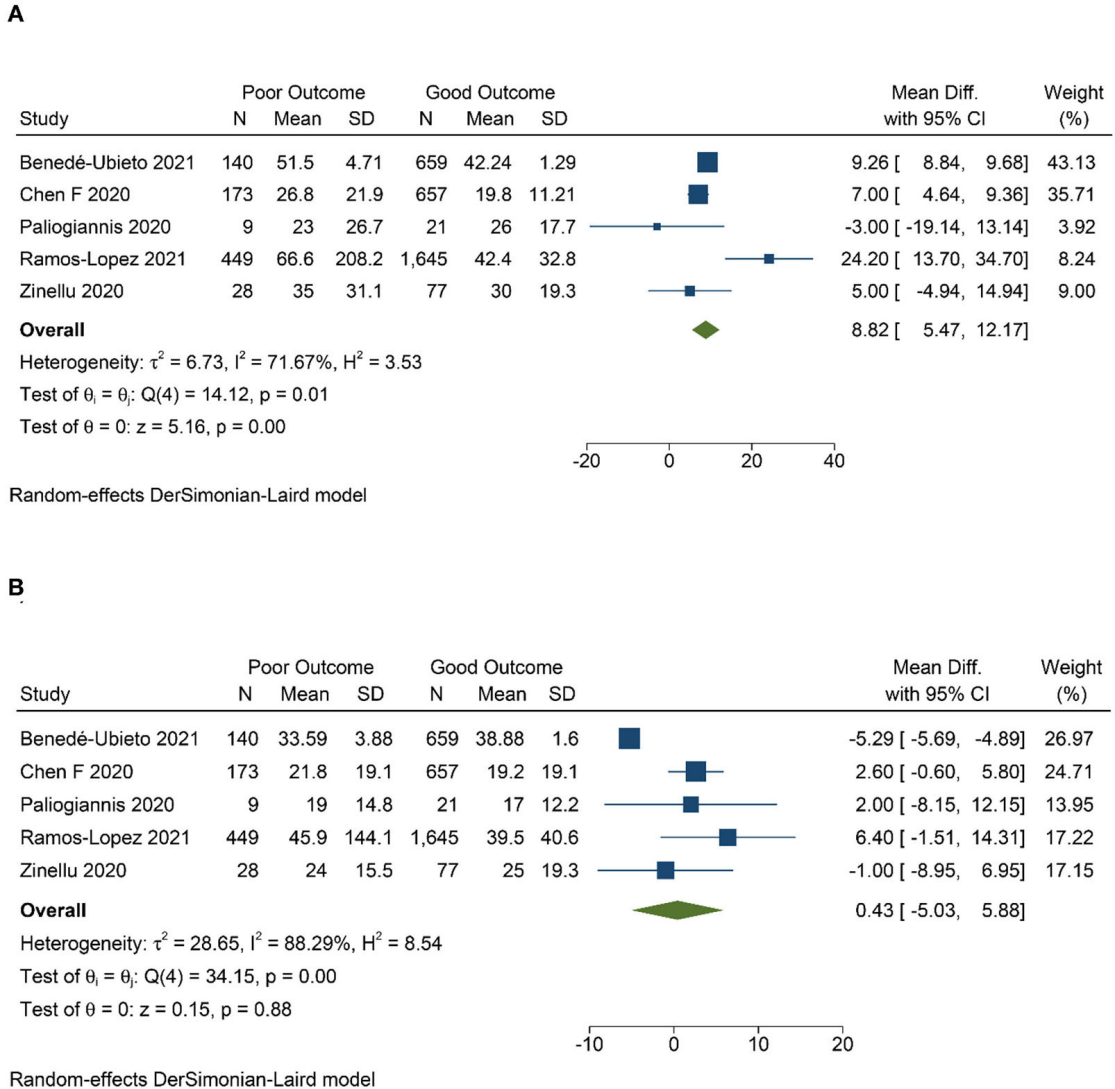
Mean difference in aspartate aminotransferase (AST) **(A)** and alanine transaminase (ALT) **(B)** level between poor and good prognosis.

There were eight studies with 4,606 patients in this meta-analysis (Figure 1) (7, 16–19). The mean age of patients in this study was 64.3 years, whereas 46.3% of the patients were male. The characteristics of the studies are presented in Table 1. Patients with poor prognosis have higher AST levels [mean difference 8.82 (5.47, 12.17), *p* < 0.001; *I*^2^: 71.7%, *p* = 0.007] (Figure 2A), but not ALT levels [mean difference 0.43 (−5.03, 5.88), *p* = 0.878; *I*^2^: 88.3%, *p* < 0.001] (Figure 2B). De Ritis ratio was elevated in 24% of the patients. Poor prognosis occurs in 26% of the patients.

**Table 1:** Characteristics of the included studies.

**Authors**	**Study design**	**Study origin**	**Cut-off for elevated De Ritis**	**Samples**	**Age (mean)**	**Male (%)**	**Obesity**	**Diabetes (%)**	**Elevated liver enzymes (%)**	**Outcome**	**NOS**
Benedé-Ubieto (20)	RC	Spain	NA	799	Stratified	54.7	NA	NA	NA	Mortality (17.5%)	6
Chen (18)	RC	China	>1	227	51	45.5	NA	9	4.5	Mortality (11.8%)	5
Medetalibeyoglu (17)	RC	Turkey	>1	554	66.2	58.7	BMI (29.39)	22.7	153/554 (27.6)	Severity (13.9%), Mortality (7.2%)	7
Paliogiannis (19)	RC	Italy	NA	60	71.5	60	NA	25	NA	Mortality (30.0%)	6
Qin (16)	RC	China	>1.38	567	55	43.6	NA	15	103/567 (18.2)	Mortality (11.5%)	8
Ramos-Lopez (21)	RC	Spain	1.29	2,094	66.9	39.4	NA	NA	NA	Mortality + ICU (21.4%)	8
Yadlapati (22)	RC	United States	1.2	200	66.5	45.5	50	NA	110/200 (55)	Mortality (26%)	6
Zinellu (7)	RC	Italy	>1.63	105	72	66.7	21.9	21	51/105 (48.6)	Mortality (26.7%)	9

*BMI, body mass index; NOS, newcastle-ottawa scale; RC, retrospective cohort*.

### Elevated De Ritis Ratio and Poor Prognosis

**Figure 3:**
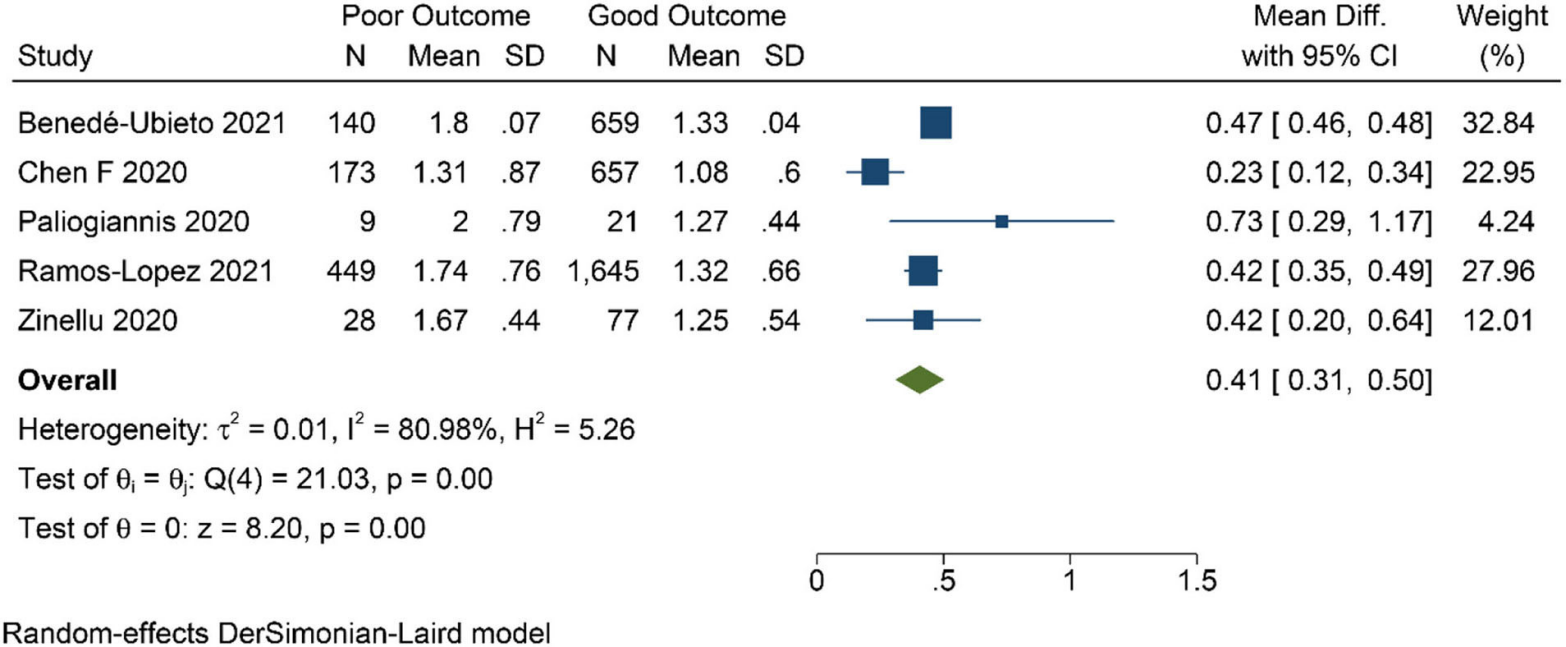
Mean difference in De Ritis ratio between poor and good prognosis.

**Figure 4:**
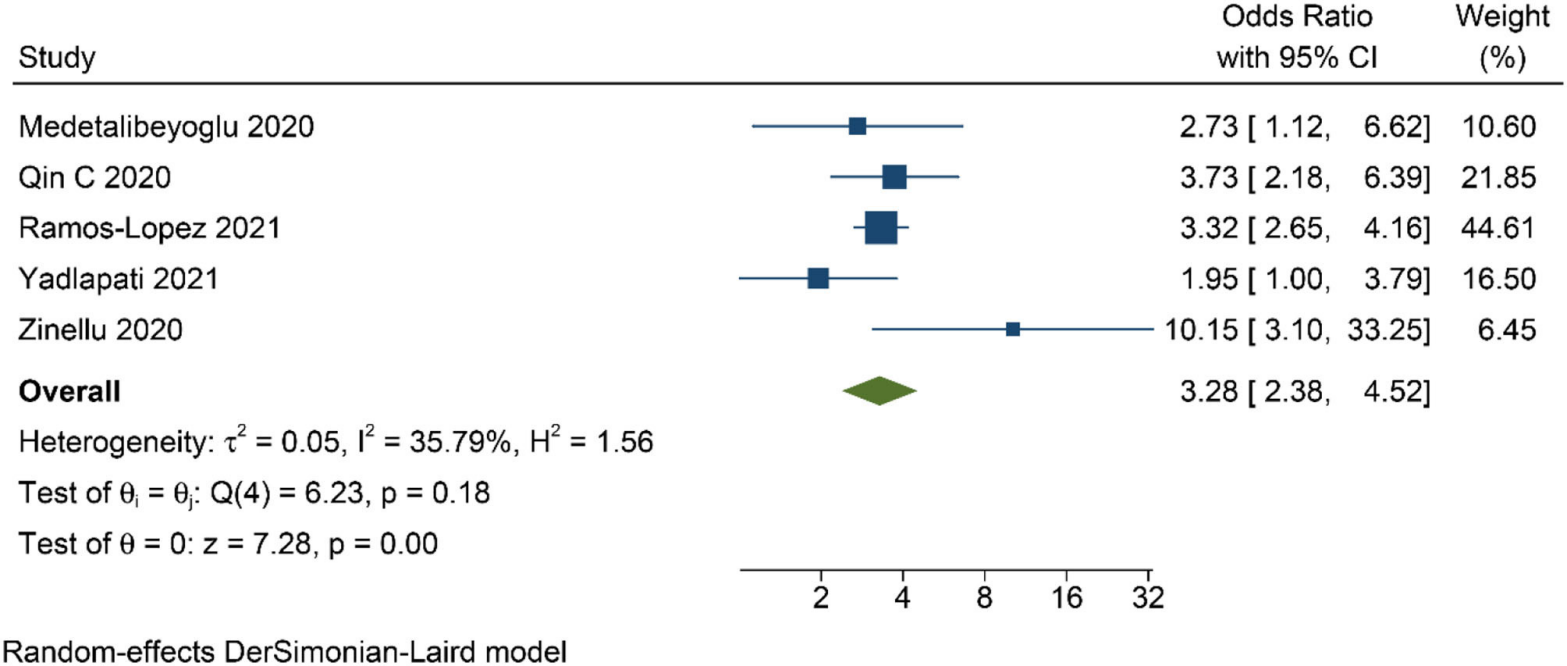
Odds ratio for elevated De Ritis ratio and poor prognosis.

**Figure 5:**
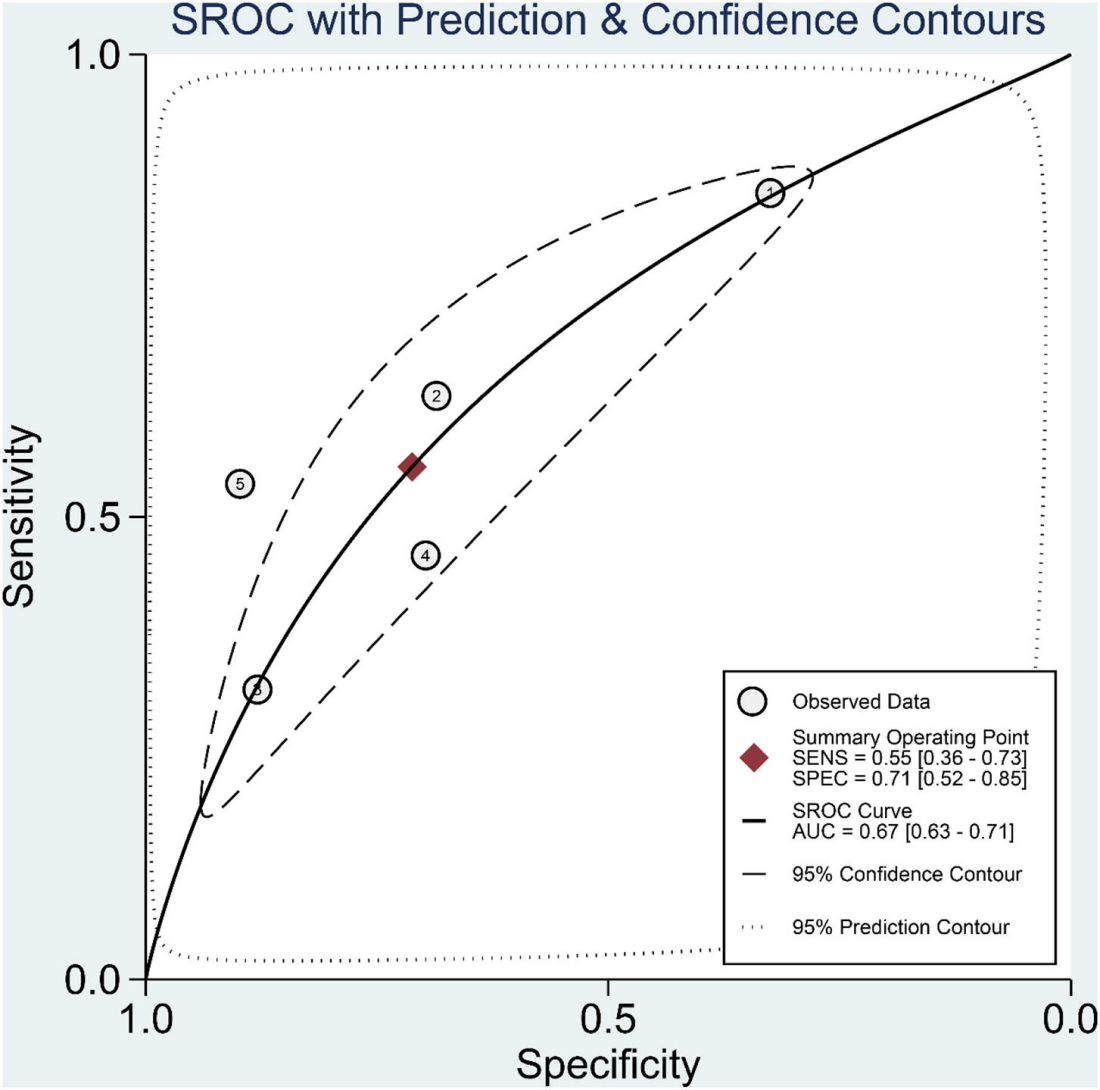
Summary receiver operating characteristic (SROC) curve.

**Figure 6:**
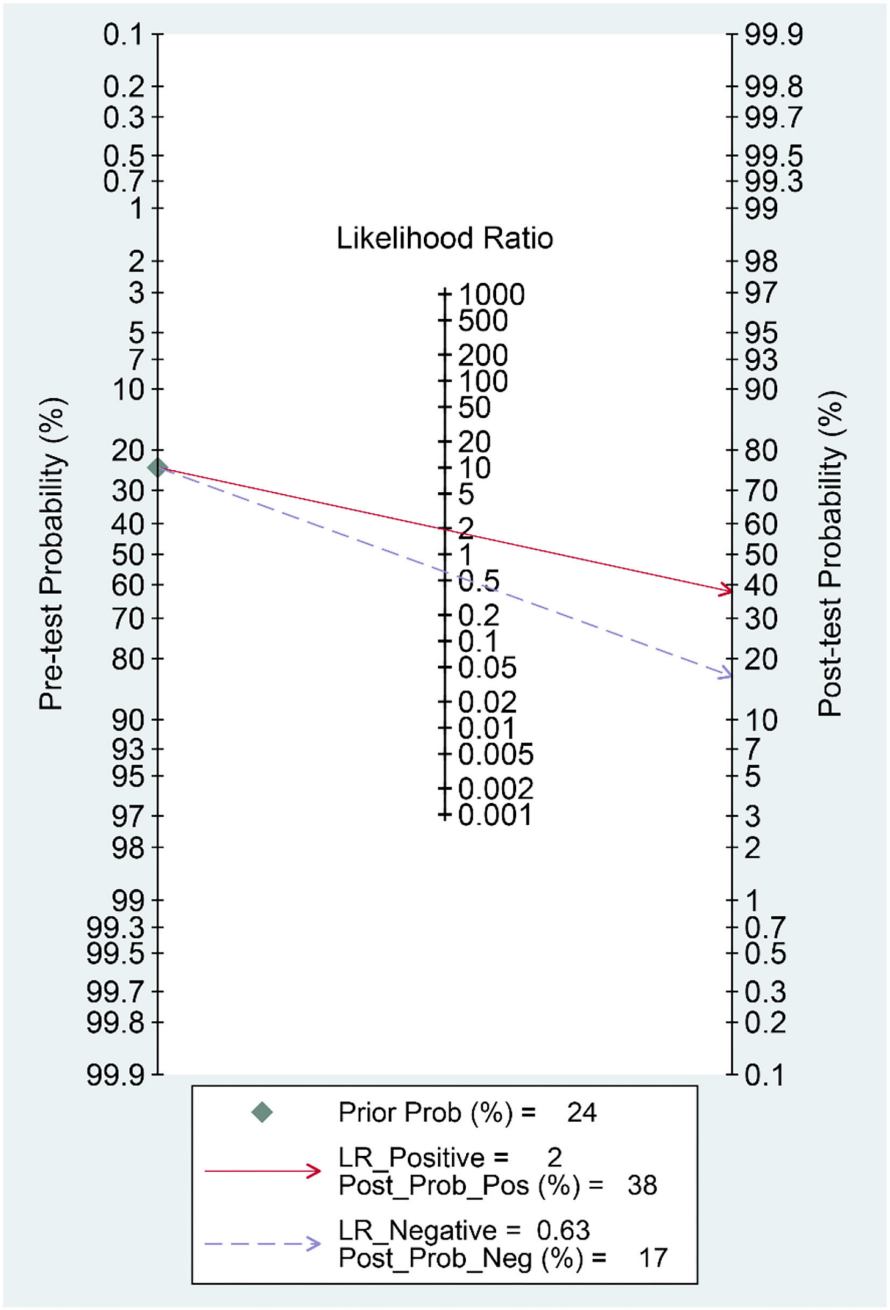
Fagan's nomogram.

Patients with poor prognosis have higher De Ritis ratio [mean difference 0.41 (0.31, 0.50), *p* < 0.001; *I*^2^: 81.0%, *p* < 0.001] (Figure 3) and subgroup analysis showed that non-survivors also have higher De Ritis Ratio [mean difference 0.47 (0.46, 0.48), *p* < 0.001; *I*^2^: 0%, *p* = 0.463]. Elevated De Ritis ratio was associated with poor prognosis [OR 3.28 (2.39, 4.52), *p* < 0.001; *I*^2^: 35.8%, *p* = 0.182] (Figure 4) and subgroup analysis also showed that elevated De Ritis ratio was associated with mortality [OR 3.36 (1.93, 5.85), *p* < 0.001; *I*^2^: 51.7%, *p* = 0.102]. It has a sensitivity of 55% (36–73), specificity of 71% (52–85), PLR 1.9, NLR 0.63, DOR of 3 (2–4), and AUC of 0.67 (0.63–0.71) (Figure 5). The posterior probability of poor prognosis was 38% if De Ritis was elevated, while 17% if De Ritis was not elevated (Figure 6).

### Risk of Bias Assessment

Newcastle-Ottawa Scale (NOS) indicates a low-moderate risk of bias (Table 1). There is no indication of small-study effects in the relationship between elevated De Ritis ratio and poor prognosis (*p* = 0.488). Deek's funnel plot asymmetry test was non-significant (*p* = 0.81).

## Discussion

Early identification of patients at risk for developing severe COVID-19 is crucial during the pandemic. Previous studies highlighted that individuals with advanced age, high body mass index, and physical inactivity had greater morbidity and mortality from COVID-19, along with the presence of various comorbidities, such as cardiovascular disease, diabetes, chronic obstructive pulmonary disease, hypertension, and chronic kidney disease (23–31). Several inflammatory parameters, comprising C-reactive protein, D-dimer, procalcitonin, interleukin-6, and ferritin, are often higher in patients with severe and critically ill with COVID-19 (8). An increase in liver-related biomarkers, particularly AST, ALT, total bilirubin concentrations, and gamma-glutamyl transferase in patients with COVID-19 have been reported (32, 33).

Although hepatic damage is not commonly seen as a major characteristic of COVID-19, liver injury is an emerging concern because it may indicate a severe disease course (2). The mechanism for liver involvement in COVID-19 remains obscure. Previous liver pathology reports showed the presence of moderate microvesicular steatosis along with mild inflammation in several areas (34). These patterns are also observed in drug-induced liver injury and sepsis, although these findings are not unique, they might provide insight into the mechanism involved in liver injury induced by COVID-19 (35). The SARS-CoV-2 may invade the liver directly through the angiotensin receptor enzyme 2 (ACE2) receptor, which serves as the novel coronavirus' entry point. It has been found that bile duct epithelial cells (cholangiocytes) express a high amount of ACE2 receptors (36). Liver dysfunction may also be caused by drug-induced liver injury or an overactive inflammatory response, including cytokine storm and pneumonia-associated hypoxia (2, 7). Antivirals used in the treatment of COVID-19 are postulated to cause drug-induced liver injury (37).

Serum concentrations of ALT and AST, without exception, are the most frequently ordered liver panel for evaluating liver injury in all laboratories. ALT is present in the cytosol of hepatocytes, while AST is present in the cytoplasm and mitochondria of the hepatocyte (38). ALT activity in the liver is ~10-fold higher than that of the heart and skeletal muscles, which emphasizes its function to indicate parenchymal liver disease or injury. Meanwhile, AST has the greatest activity in the liver, cardiac, and skeletal muscle, but also exhibits in other tissues including kidneys, pulmonary, brain, pancreas, red blood cells, and white blood cells. Therefore, ALT is a more specific biomarker for liver damage compared to AST, indicating liver-biliary disease, myocardial injury, and rhabdomyolysis (7, 15). AST and ALT are found in the liver with a 2.5:1 ratio but with different turnaround time, resulting in a relatively similar level of serum of AST and ALT in healthy populations (38).

The De Ritis ratio or the AST/ALT ratio is a promising biochemical parameter for prognostication in COVID-19. In the present study, elevated De Ritis ratio was associated with 3-fold increased risk for poor prognosis in patients with COVID-19. Although the cut-off values for elevated De Ritis ratio are different from these five studies (Table 1), the result of this meta-analysis has low heterogeneity (*I*^2^: 35.8%). Nonetheless, the difference in the cut-off value used between those studies caused a highly varied diagnostic value (Figure 3) with an overall sensitivity of 55%, specificity of 71%, and AUC of 0.67. These variations further translate into the uncertainty of the optimal cut-off value for De Ritis Ratio as a prognostic factor in COVID-19 and merit further investigations.

Interestingly, Qin et al. indicated that De Ritis ratio of ≥1.38 was independently associated with poor prognosis irrespective of AST elevation (≤40 and >40 U/L) (16). They showed that AST/ALT ratio elevation was associated with a more severely computed tomography scan findings, higher severity, and positive linear association with other prognostic markers (e.g., c-reactive protein, procalcitonin, interleukin-6, D-Dimer, lactate, LDH, and creatine Kinase-MB). Additionally, Chen et al. showed the association of AST/ALT ratio with liver injury and severity of COVID-19. However, the number of outcomes or risk estimates (e.g., OR) of this study interest was not available (18).

There were two studies on the association of De Ritis ratio with other specific biochemical parameters (e.g., creatinine kinase and serum ALT), but were excluded from the analysis due to its irrelevance with our outcome of interest (15, 39).

The limitations of the current study were primarily caused by the small quantity of the included studies. Moreover, the retrospective-observational nature and the small sample size of the included studies should be taken into account in extrapolating the results of this meta-analysis, where selection bias and confounding factors may be evident. We also could not dismiss the possibility of publication bias due to the small number of studies. Despite our limitations, this meta-analysis has brought early evidence of using the De Ritis ratio for prognostication in COVID-19.

### Implication for Clinical Practice

Although this “traditional” ratio was initially found in 1957 as a diagnostic test for viral hepatitis (40), it is still commonly used and proves to be a valuable indicator of liver disease (38). It is a promising, straightforward, and readily available parameter for poor prognosis in COVID-19. This meta-analysis showed that AST, but not ALT, was significantly associated with poor prognosis in COVID-19. This supports the use of De Ritis ratio in addition to AST and ALT levels. However, we suggest, including this parameter and other accessible hematological markers, to improve the prognostic performance of the model for COVID-19. De Ritis ratio would be better for this marker to be a part of a prognostic model rather than a stand-alone examination. A predictive model comprising of readily available tools may be of value, especially in rural areas where sophisticated prognostic biomarkers are often not available.

In conclusion, elevated De Ritis ratio is associated with poor prognosis in patients with COVID-19.

## Data Availability Statement

The raw data supporting the conclusions of this article will be made available by the authors, without undue reservation.

## Author Contributions

RP: conceptualization, methodology, formal analysis, investigation, and writing—original draft. IH: data curation, investigation, writing—original draft, and project administration. ML: data curation, investigation, and writing—original draft. EY and RV: investigation and writing—original draft. AL, SN, BS, and RK: investigation and writing—review and editing. All authors contributed to the article and approved the submitted version.

## Conflict of Interest

The authors declare that the research was conducted in the absence of any commercial or financial relationships that could be construed as a potential conflict of interest.

## Publisher's Note

All claims expressed in this article are solely those of the authors and do not necessarily represent those of their affiliated organizations, or those of the publisher, the editors and the reviewers. Any product that may be evaluated in this article, or claim that may be made by its manufacturer, is not guaranteed or endorsed by the publisher.

## Abbreviations

ACE2: Angiotensin receptor enzyme 2
AST: Aspartate aminotransferase
ALT: Alanine aminotransferase
AUC: Area under curve
COVID-19: Coronavirus disease 2019
DOR: Diagnostic odds ratio
OR: Odds ratio
PLR: Positive likelihood ratio
NLR: Negative likelihood ratio
SARS-CoV-2: Severe cute respiratory syndrome coronavirus 2.
